# A scale‐down mimic for mapping the process performance of centrifugation, depth and sterile filtration

**DOI:** 10.1002/bit.25967

**Published:** 2016-03-16

**Authors:** Adrian Joseph, Brian Kenty, Michael Mollet, Kenneth Hwang, Steven Rose, Stephen Goldrick, Jean Bender, Suzanne S. Farid, Nigel Titchener‐Hooker

**Affiliations:** ^1^ The Advanced Centre of Biochemical Engineering, Department of Biochemical EngineeringUniversity College London, Bernard Katz BuildingLondonWC1E 6BTUnited Kingdom; ^2^ MedImmune LLC Gaithersburg Headquarters, One MedImmune WayGaithersburgMaryland

**Keywords:** centrifugation, continuous centrifugation, scale‐down, primary recovery, mammalian cell, disk‐stack centrifuge, depth filter, capillary shear, filter capacity

## Abstract

In the production of biopharmaceuticals disk‐stack centrifugation is widely used as a harvest step for the removal of cells and cellular debris. Depth filters followed by sterile filters are often then employed to remove residual solids remaining in the centrate. Process development of centrifugation is usually conducted at pilot‐scale so as to mimic the commercial scale equipment but this method requires large quantities of cell culture and significant levels of effort for successful characterization. A scale‐down approach based upon the use of a shear device and a bench‐top centrifuge has been extended in this work towards a preparative methodology that successfully predicts the performance of the continuous centrifuge and polishing filters. The use of this methodology allows the effects of cell culture conditions and large‐scale centrifugal process parameters on subsequent filtration performance to be assessed at an early stage of process development where material availability is limited. Biotechnol. Bioeng. 2016;113: 1934–1941. © 2016 The Authors. *Biotechnology and Bioengineering* Published by Wiley Periodicals, Inc.

**Abbreviations**
CSD: capillary shear device
RSD: rotating shear device
USD: ultra scale down
LDH: lactate dehydrogenase
mAb: monoclonal antibody
NADH: nicotinamide adenine dinucleotide
PEEK: polyetheretherketone
QbD: quality by design

## Introduction

Quality by Design (QbD) regulatory initiatives over the last decade have required that biopharmaceutical manufacturers develop a thorough understanding of a product's quality attributes and manufacturing process through the generation of design spaces (Rathore, 2009). High throughput scale‐down techniques now enable the rapid generation of extensive experimental data representative of large‐scale performance, both in the upstream and downstream manufacturing process. Such large experimental data sets generated through these techniques allow for better identification of the effects and interactions of the input parameters on the process performance and product quality (Titchener‐Hooker et al., 2008).

Continuous flow disk‐stack centrifugation is often used to harvest commercial‐scale cell culture processes because of its robustness and relatively low running costs (Axelsson, 2002). The intermittent discharge provided by the disk‐stack centrifuge enables removal of a considerable quantity of cells and large cellular debris in a semi continuous fashion. However, one of the disadvantages of disk‐stack centrifugation is that in many designs the cells enter the centrifuge through a feed zone in which high levels of shear are present. This shear can damage shear‐sensitive mammalian cells resulting in the generation of submicron particles which are carried over to the centrate (Jain et al., 2005). These fine particles can cause subsequent fouling in later chromatographic processes, resulting in high column pressures and accompanied reductions in column lifetime and efficiency (Kempken et al., 1995). In order to avoid chromatographic column fouling, depth filtration is often used immediately after centrifugation to remove these submicron particles (Yigzaw et al., 2006). Hence, a typical process sequence for a mammalian cell culture process might begin with the removal of cells and cell debris achieved through a combination of a centrifugal step followed by a depth filtration step (Shukla and Kandula, 2008).

In order to scale among different types of centrifuges, correction factors are used to account for the deviations from ideal conditions such as those caused by differences in flow patterns (Mosqueira et al., 1981). Typically, Sigma theory is used to scale centrifuges irrespective of size, geometry, and type (Ambler, 1959). However, Sigma theory does not take into account the generation of small particles through cell damage in the high shear regions of centrifuge feed zones. In order to capture accurately these effects at laboratory‐scale, the shear generated in the feed zone needs to be mimicked (Boychyn et al., 2001). A Rotating Shear Device (RSD) has been developed to reproduce the prevailing shear conditions in such feed zones (Boychyn et al., 2001). Operated in combination with a bench‐top centrifuge, this has been shown to successfully predict the clarification efficiency of mammalian cell culture in a pilot‐scale disk‐stack centrifuge (Hutchinson et al., 2006). The RSD has also been used in conjunction with micro well plates using sub‐millilitre volumes to successfully model pilot‐scale centrifugation performance (Tait et al., 2009).

In the routine development of primary recovery operations, the performances of both centrifugal and filtration processes need to be characterized. The RSD was developed as an ultra scale‐down (USD) tool to investigate under well‐controlled and ‐defined conditions using small quantities of feedstock. Extensive publications have shown the utility of the RSD to understand the impact of exposure to various levels of shear as might be experienced in the centrifugal step at pilot to manufacturing scale (Boychyn et al., 2001; Hutchinson et al., 2006; Tait et al., 2009). However, in order to test secondary depth filter capacity, large quantity (≈1 L) of centrate is required. The RSD is limited to shearing a maximum of 20 mL of material per run hence restricting its ability to provide the quantity of feedstock needed to characterize subsequent filtration performance. The Capillary Shear Device (CSD) has also shown to be a preparative device with the ability to mimic feed zone shear. Flow through the capillary enables the generation of energy dissipation rates (EDR) equivalent to those found in disk‐stack centrifuges (Westoby et al., 2011). Furthermore, this methodology has shown that it can generate centrates with a particle size distribution equivalent to that from a large‐scale centrifuge (Westoby et al., 2011). In theory, such a device can be used to produce unlimited quantities of sheared material. To date no comparison between the characteristics of the material prepared by the CSD and the established RSD has been published.

In this paper, a combination of scale‐down devices was used to explore the impact of centrifugal separation on filter capacity so as to determine the best integration between the steps of centrifugal solids removal and submicron particulate elimination by depth filtration ahead of packed bed high resolution steps of purification. It explores the utility of the CSD to create a pool of sheared feed material for centrifugal separation and the creation of a realistic centrate pool for the subsequent characterization of the following depth filtration step. In this sense, it is proposed to use the CSD as a preparatory method for filtration studies greater than 1 L or more of feed material. Critical to the success of this approach is the quality of the CSD material. The process characteristics and properties of the CSD preparatory scale material are compared against the RSD as a proven method for scale‐down studies.

## Materials and Methods

### Theoretical Considerations

A detailed discussion of scale‐down centrifugation is given in prior studies (Boychyn et al., 2001; Hutchinson et al., 2006). In essence by maintaining the ratio of flow rate to equivalent settling area and accounting for deviations from ideal flow conditions, Sigma theory enables the direct comparison of clarification efficiencies between centrifuges of different sizes and geometries (Ambler, 1959). Hence, this methodology can be applied to predict the clarification performance of a disk‐stack centrifuge using a laboratory bench‐top centrifuge:

(1) $$\frac{{\;}_{Q_{ds}}}{c_{ds}\Sigma_{ds}}=\frac{{\;}_{V_{lab}}}{t_{lab}c_{lab}\Sigma_{lab}},$$

where *Q*_ds_ is the volumetric flow rate into the disk‐stack centrifuge, *V*_lab_ is the volume of material used in the laboratory centrifuge, *t*_lab_ is the centrifugation time, and *c*_ds_ and *c*_lab_ are correction factors that account for the non‐ideal flow properties in the disk‐stack and laboratory scale centrifuges, respectively. The correction factor for the disk‐stack centrifuge is quoted to be ≈0.4 by (Pinheiro and Cabral, 1993) while the correction factor for the laboratory scale centrifuge is 1. Here, the relationship determined by Maybury et al. (2000) is used to describe the equivalent settling area of a laboratory‐scale centrifuge (*Σ*_lab_):

(2) $$\Sigma_{lab}=\frac{V_{lab}\omega^{2}\left(3-2x-2y\right)}{6g\ln\left({2R}_{2}/\left(R_{2}+R_{1}\right)\right)},$$

*ω* is the angular velocity of the rotor; *x* and *y* are the fractional acceleration and deceleration times. *R*_1_ is the distance from the top of the liquid level of the centrifuge tube to the center of the laboratory‐scale centrifuge's axis of rotation, while *R*_2_ is the distance from the bottom of the centrifuge tube to the center of the axis of rotation. The equivalent settling area for a disk‐stack centrifuge (*Σ*_ds_) is described by:

(3) $$\Sigma_{ds}=\frac{2}{3g}\pi \;z\;\omega^{2}\;\cot\;\theta \left({r_{2}}^{3}-{r_{1}}^{3}\right),$$

where *z* is the total number of disks, *θ* is the half disk angle, *r*_1_ and *r*_2_ are the inner and outer disk radii, respectively.

### Cell Culture

Cell culture used in the experiments were generated using CHO cell lines expressing monoclonal antibody products. The cultures produced had a range of cell densities and viabilities measured at the day of harvest as summarized in Table I. Bench‐scale cell culture was conducted in 3 L fed batch bioreactors while pilot‐scale culture was performed in 50, 100, and 2000 L stainless steel bioreactors. All cultures are harvested between days 11 and 14 during the decline phase of growth.

**Table I:** Cell culture properties

Material	Bio reactor size (L)	Cell density ×10^6^ (cells/mL)	Cell viability (%)
Culture‐A	100	24.5	68
Culture‐B	100	26.8	58
Culture‐C	2000	14.9	74
Culture‐D	2000	16	70
Culture‐E	2000	12.2	45
Culture‐F	3	30.8	31
Culture‐G	50	11.6	72

### Rotating Shear Device (RSD)

The design, theory, and application of the rotating shear device to mimic levels of shear found in centrifuge feed zones has been thoroughly covered in previous studies (Boychyn et al., 2004; Hutchinson et al., 2006; Maybury et al., 2000). The material to be sheared is held in a cylindrical chamber with a diameter of 50 mm and a height of 10 mm. A rotating disk located within the chamber creates the shear and has a diameter of 40 mm and a thickness of 1 mm. The correlation of the rotating speed of the disk to the levels of energy dissipation has been developed in previous studies (Boychyn et al., 2004). In the experiments conducted for this study, the rotating shear device was run at a range of speeds in order to identify the equivalent shear generated in both the Capillary shear device (CSD) and the pilot‐scale centrifuge. Material was subjected to shear in the chamber for 20 s to ensure that the total content was equally exposed to shear generated by the rotating disk (Hutchinson et al., 2006).

### Capillary Shear Device (CSD)

**Figure 1:**
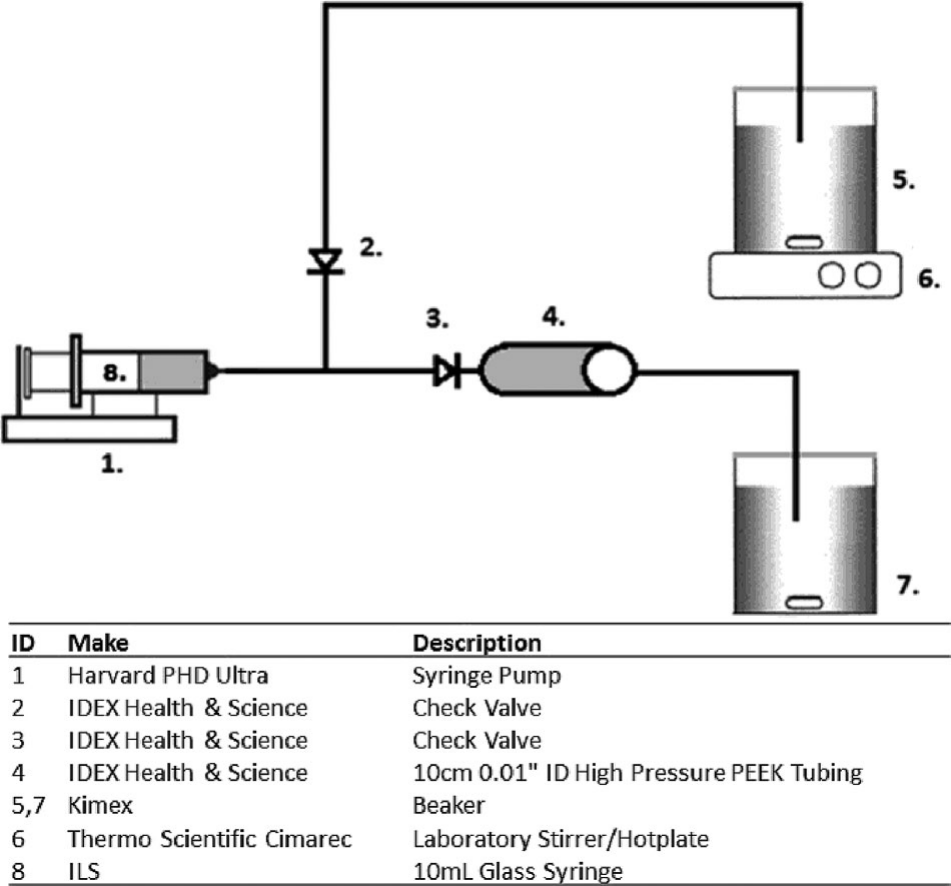
Preparative CDS apparatus schematic and parts list.

The use of capillary based shear systems to mimic levels of energy dissipation experienced in disk‐stack centrifugal processes have been extensively described in literature (Aucamp et al., 2014; Chan et al., 2006; Westoby et al., 2011). The CSD described in this publication consisted of a Harvard syringe pump (Cambridge, UK), 0.01″ ID 10 cm long Polyetheretherketone (PEEK) tubing, 10 mL glass syringe, and check valves as set out in Figure 1. The cell culture was delivered from a vessel to the glass syringe via a check valve (ID‐2). Subsequently, the flow through the capillary was achieved through another check valve (ID‐3) with the syringe pump in infuse mode. Varying the level of shear was achieved by adjusting the flow rate through the capillary. This relationship has been developed in more detail in previous studies (Ma et al., 2002; Mollet et al., 2007, 2004). The culture was kept well mixed using a stir plate and stir bar. The sheared material was collected in a sealed vessel.

### Identification of Pilot‐Scale Centrifuge Shear

In order to identify the levels of shear at a range of different flow rates through the CSD, the lactate dehydrogenase (LDH) concentration of the cell culture was measured before and after shearing. The relationship between the level of LDH increase as a function of flow rate (Q) was established and used to calculate the capillary flow rate that resulted in an equivalent level of cell lysis in the preparative CSD to that of the standard ultra scale‐down RSD. This provided the necessary bridging study between the RSD, preparative CSD, and the pilot‐scale centrifuge.

### Identification of Pilot‐Scale Centrifuge Separation Efficiency

Correction factors of ≈0.4 have been used to describe industrial disk‐stack centrifuges; however, not all centrifuges behave identically. To estimate the correction factor of the pilot‐scale centrifuges examined in this study, material was generated using the preparative CSD under conditions which corresponded to the level of shear observed in the centrifuge as verified by the RSD data. Subsequently, the sheared material was centrifuged in a bench top centrifuge at a range of V/tΣ. The supernatants from the range of centrifuged samples were analyzed for the extent of solids remaining. This data set was used to identify the best value for the effective centrifuge correction factor.

### Centrifuge Experiments and Protocols

#### Floor Swing‐Out Rotor Centrifuge

In order to generate large quantities of centrate required for depth filtration experiments, it was necessary to explore the preparative characteristics of the CSD. Immediately following exposure to capillary shear, the sheared cell culture material was placed into a series of 50 mL polypropylene tubes and centrifuged at 2600*g* with varying liquid height and for varying durations in order to generate the required range of V/tΣ. Centrifugation was conducted in a Beckman J‐HC centrifuge with a JS 4.2A rotor (Brea, CA). The Optical Density (OD) of the supernatant was measured following each run at a wavelength of 600 nm.

#### Pilot‐Scale Centrifuge

Continuous flow Alfa Laval LAPX‐404 and BTPX‐305 disk‐stack centrifuges (Lund, Sweden) were used to clarify the cell culture at large‐scale. In the experiments conducted with the LAPX‐404 flow rates were set at 90–120 L/h and the rotational speed of the bowl was varied to generate between 6000 and 10,000 g while the larger BTPX‐305's flow rate and bowl speed were maintained at 480 L/h and 12,500 g, respectively.

### Depth and Sterile Filtration Protocols

The centrate clarification experiments were conducted using a Millistak+ X0HC depth filter (EMD Millipore, Billerica, MA) with a nominal pore size ranging from 0.1 to 2 μm. Sterilizing grade SHC filters (EMD Millipore) were used to filter the depth filtered material. The SHC has a bilayer structure with a pore size rating of 0.5/0.2 μm.

Depth filtration capacity (L/m^2^) was identified by observing the change in filter pressure at a constant flow rate. This approach is known as the *P*_max_ methodology (Yavorsky et al., 2003). In this study, *P*_max_ was defined as the capacity at which a pressure drop of 10 psi was reached. The experimental runs were conducted using a 23 cm^2^ filter capsule at 200 LMH. Pressure drops and filtrate turbidities (NTU) were recorded for the duration of the experiment. In cases where the feed pool of centrate had been exhausted and a pressure of 10 psi had not been attained an intermediate pore blockage model was linearized (Eq. (4)) to predict theoretically the volume of filtrate per filter area (*V*) required to reach a pressure (Δ*P*) of 10 psi (Hlavacek and Bouchet, 1993). In the linearized equation, *a*″ and *b*″ were used as dimensionless coefficients.

(4) $$\ln\left(\Delta P\right)=a^{\prime \prime }V+b^{\prime \prime },$$

The capacity of SHC sterile filters required to filter the X0HC filtrate was established through the *V*_max_ methodology (Badmington et al., 1995). Experiments were conducted with a 3.5 cm^2^ at a constant pressure of 10 psi and the flux decline was monitored over time. The *V*_max_ method is a linearized form (Eq. (5)) of the pore constriction model used to compute theoretically the maximum volume of filtrate per unit filter area (*V*_max_) that can be obtained before complete fouling through plugging occurs (Kong et al., 2010; Lau et al., 2013; Zydney and Ho, 2002).

(5) $$\frac{t}{V}=\frac{1}{Q_{0}}+\left(\frac{1}{V_{\max}}\right)t,$$

where *V* is the total volume of filtrate volume per unit filter area collected over time (*t*) and *Q*_0_ is the initial specific volumetric filtrate flow rate per unit filter area.

### Solids Remaining

The performance of a centrifuge in removing solids at any given set of operating conditions may be described by calculating the percentage of solids remaining in the supernatant (*S*).

(6) $$S=(\frac{{OD}_{s}-{OD}_{o}}{{OD}_{f}-{OD}_{o}})\times 100\%,$$

where OD*_s_* is the filtrate clarity post depth filtration, OD*_o_* is the base line for a well clarified centrate (obtained by extended centrifugation for 30 min at 16,000*g* (Tait et al., 2009)) OD*_f_* refers to the clarity of the feed stream prior to depth filtration. All optical densities were measured at 600 nm.

### Characterization of Cell Lysis

Lactate dehydrogenase (LDH) is an intracellular enzyme that is only released during cell rupture. LDH increase was used to characterize the extent of cell lysis occurring during the centrifugation process (Ma et al., 2002; Petersen et al., 1988). It was also used to establish comparability between the established scale‐down method for generating defined levels of shear (RSD) and the preparative CSD method explored in this study for the generation of filter feed volumes. Following assay protocols provided in the BioVision Kit (Milpitas, CA) samples that had been exposed to shear in *either* device were immediately centrifuged at 10,000*g* for 15 min for the separation of cell debris from the sample. The supernatant was removed and combined with Nicotinamide adenine dinucleotide (NADH) and pyruvate solutions. The sample mixture was then aliquoted into a microwell plate and a microplate reader used to measure the absorbance change. The LDH increase (LDH_INC_) was used calculated by dividing the change in LDH activity in the non‐sheared sample (LDH_NS_) and sheared sample (LDH_NS_) by that of the non‐sheared sample.

(7) $${LDH}_{INC}=\left(\frac{{LDH}_{SS}-{LDH}_{NS}}{{LDH}_{NS}}\right)\times 100\%$$

## Results and Discussion

**Figure 2:**
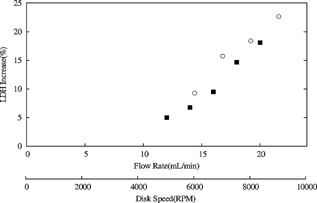
Comparison of LDH increase for Culture‐A (Table I) sourced centrates generated using the preparative scale capillary shear device (CSD, ▪) and rotating shear device (RSD, 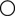). The ranges tested for the rotational speed and flow rate incorporate literature values that have been shown previously to mimic the shear damage generated in a centrifuge equipped with a hyrdo‐hermetic feed‐zone.

Extensive work has been published on the use of the Rotating shear device (RSD) to understand the consequence of shear stress placed on cell culture materials and thus predicting the impact of similar levels of exposure prevailing in the feed zones of pilot‐scale centrifuges. By the use of such a scale‐down device it has been possible to approximate the levels of energy dissipation in both hydro and non‐hydro hermetically sealed centrifuges (Boychyn et al., 2004). In this study, the need to prepare large quantities of sheared material so as to satisfy the feed requirements for subsequent filtration experiments was met by use of a Capillary shear device (CSD). Integral to this was the need to compare the product of this device to that of the RSD to demonstrate comparability in the amount of shear stress generated and of the resultant material properties. Figure 2 shows the LDH increase for a typical cell culture broth sheared using the CSD at flowrates from 12 to 20 mL/min and the RSD at rotational speeds between 6000 and 9000 RPM. A linear increase in LDH release is observed in both cases and the data from both systems overlap each other suggesting similar levels of energy dissipation can be generated using the two devices. These results encompass the flow rates reported in literature to mimic the shear in a hydro‐hermetically sealed centrifuge for operation of a CSD (Westoby et al., 2011) and the range of rotational speeds used in the RSD to achieve the same mimic (Boychyn et al., 2004).

**Figure 3:**
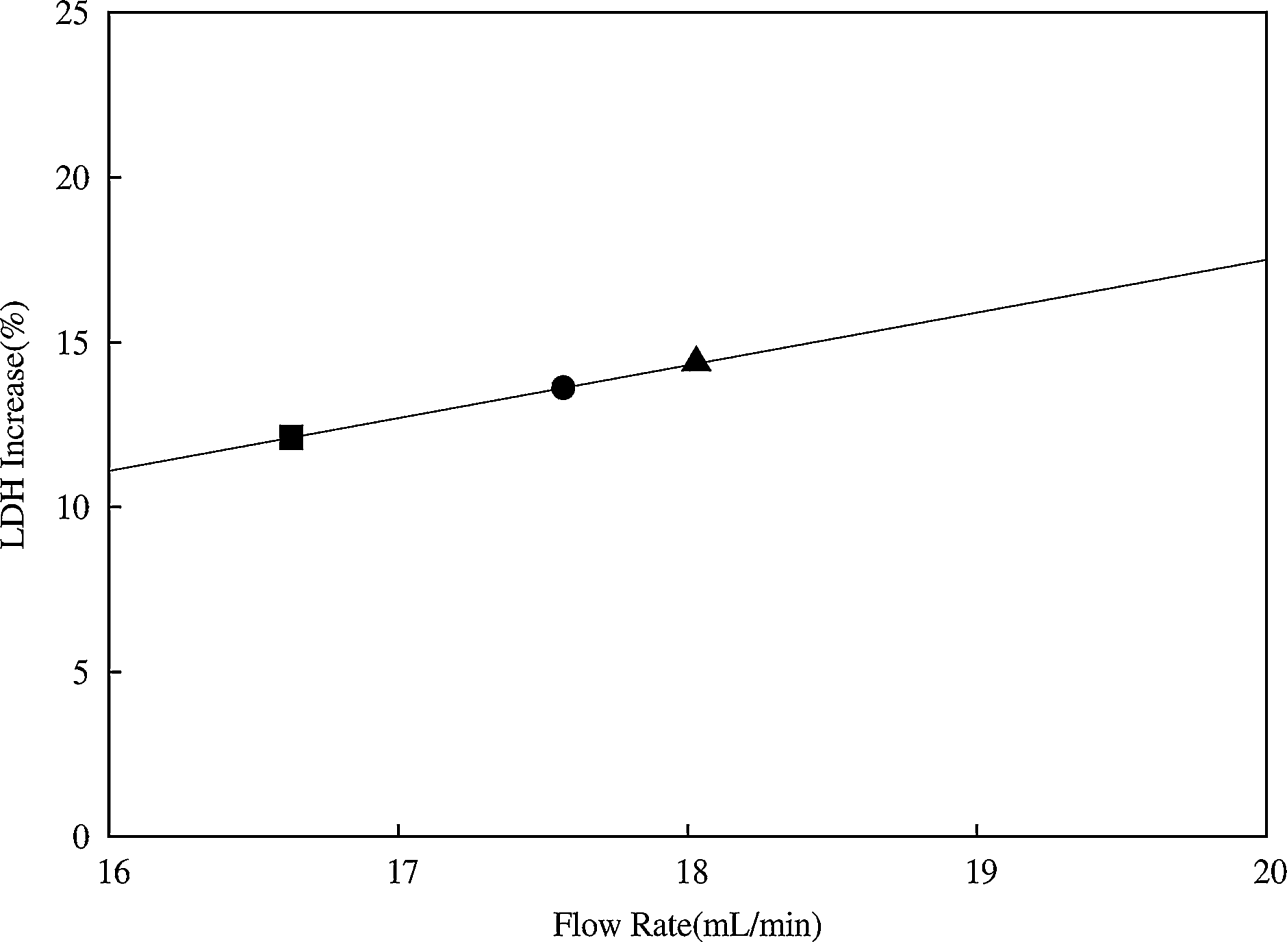
LDH increase for the modeling datasets generated by shearing material (Culture‐B(—)) (Table I) using the preparatory CSD to identify the shear in the LAPX‐404 centrate at conditions 1 (▪), 2 (●), and 3 (▴) (Table II). Regression analysis was used to determine the following relationship between LDH increase (LDH_INC_) and flowrate (Q) for Culture‐B: LDH_INC_ = 1.60 Q − 14.49, *R* ^2^ = 0.98.

Having established the comparability of the two devices, the next step was to mimic the process performances of the pilot‐scale centrifuges; Alfa Laval LAPX‐404 and BTPX‐305. The CSD was used as a proxy to approximate the levels of shear to which material is exposed inside the centrifuge at 6000, 7900, and 10,000*g* for the LAPX‐404 and at 12,500*g* for the BTPX‐305. Data shown in Figure 3 provided a correlation connecting the operating flow rate within the CSD to the conditions of operation in the LAPX‐404 centrifuge (Table II) via levels of LDH increase (LDH_INC_). Regression analysis of the modeling dataset and matching of the LDH increase showed that the levels of shear in the feed zone of the LAPX‐404 and BTPX‐305 were equivalent to the levels of shear created at a flow rate range of 16.6–18.0 mL/min in the CSD. This result is consistent with earlier published data (Westoby et al., 2011).

**Table II:** Operational settings for pilot‐scale centrifuge and corresponding CSD settings

	Pilot‐scale centrifuge			Capillary shear device (CSD)	
Condition	Centrifuge	Flow rate (L/h)	Relative centrifugal force (g)	*Q*/cΣ, *V*/tΣ × 10^−8^ (m/s)	CSD flowrate (mL/min)
1	LAPX‐404	90	6000	4.9	16.63
2	LAPX‐404	105	7900	2.92	17.57
3	LAPX‐404	120	10,000	1.65	18.03
4	BTPX‐305	480	12,500	2.2	17.08

**Figure 4:**
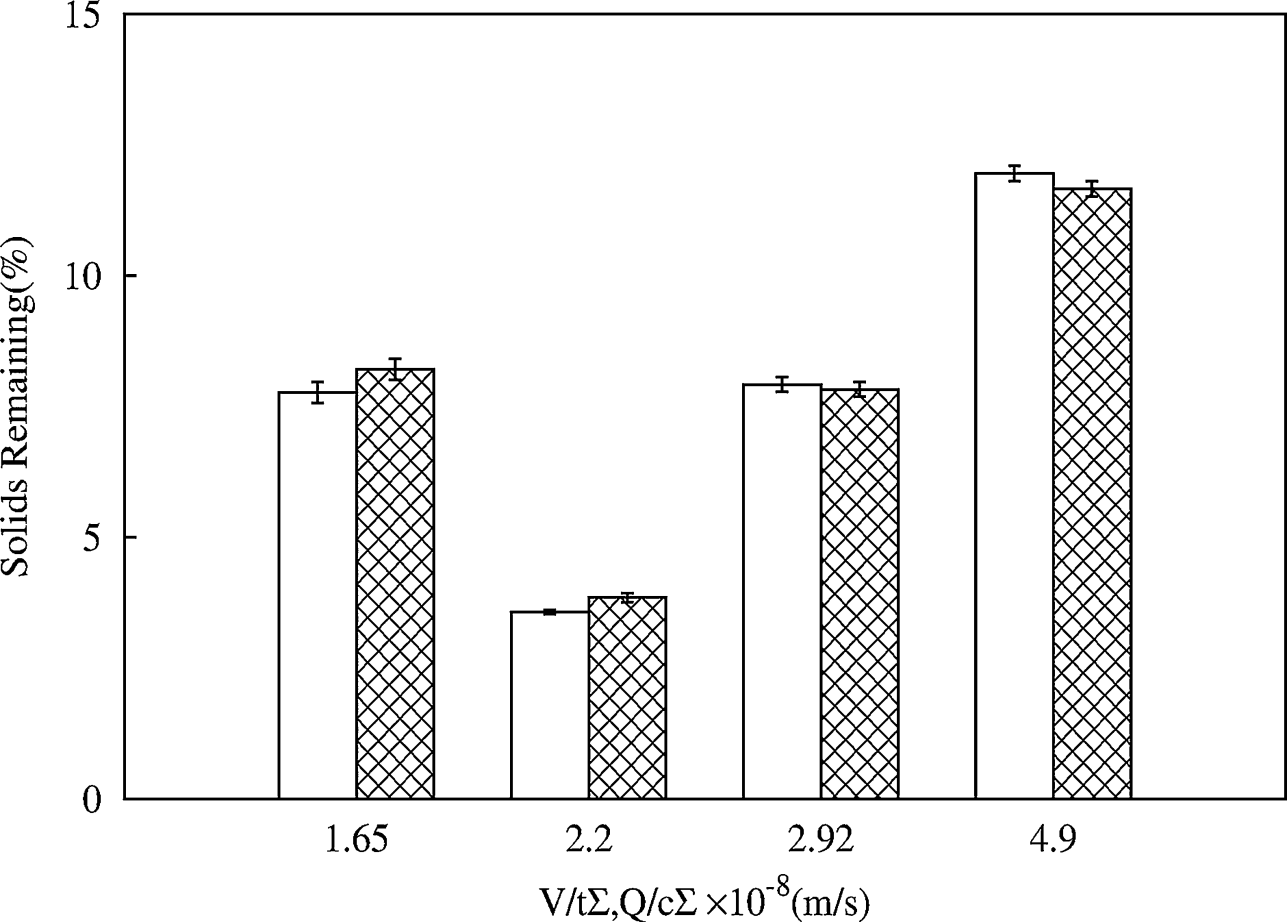
Comparison of solids remaining in centrate generated using the preparative CSD mimicking LAPX‐404 and BTPX‐305 at a range of conditions (Table II) marked in 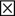 and solids remaining from pilot, scale centrifuge runs marked in □. Centrates generated for LAPX‐404 were sourced from Culture‐B (Table I) while the BTPX‐305 centrate was sourced from Culture‐C (Table I). The values plotted are shown as mean ± SD (*n* = 3).

The next step of predicting the pilot‐scale centrifuges' process performance was to identify the centrifuge correction factors. The relationship between solids remaining (S) and *V/tΣ* was empirically determined (*S* = 7.1 × ln (*V/tΣ*) − 130.3, *R* ^2^ = 0.99) and the correction faction of the Alfa Laval LAPX‐404 was found to be approximately 0.27 while a correction factor of 0.55 was established to describe the BTPX‐305 (modeling data set: *S* = 2.5 × ln (*V/tΣ*) − 48.0, *R* ^2^ = 0.99). These results and other independent studies using multiple cell culture samples determined the correction factor of the LAPX‐404 to be in the range of 0.27–0.31, confirming the correction factor values indicated were representative of the centrifuge's attributes. The preparative methodology based on the CSD was then used to mimic a set of Q/cΣ in the LAPX‐404 and BTPX‐305 machines (Table II). The solids remaining in centrates generated using the CSD closely matched the solids remaining in centrates generated by the pilot‐scale centrifuges (Fig. 4). This confirmed the validity of the approach to provide quantities of representative material for process integration studies.

**Figure 5:**
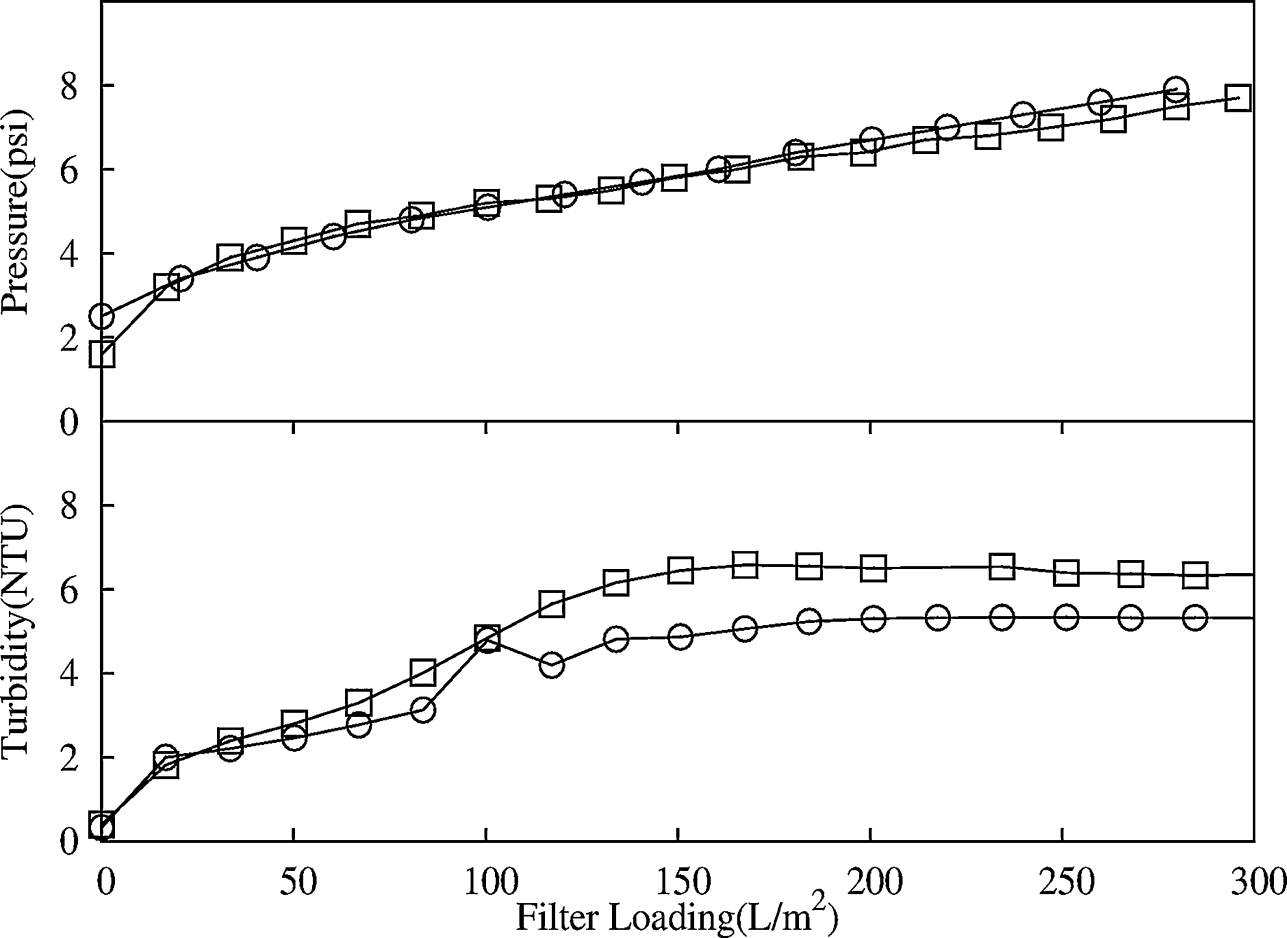
Comparison of pressure and turbidity profiles for 0.1–2.0 μm X0HC depth filter when filtering centrate from the BTPX‐305 machine (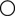) and the mimic centrate (□) generated applying the preparative methodology presented in the paper. BTPX‐305 centrate for this study was generated at a relative centrifuge force of 12,500*g*. Preparatory CSD centrate was processed to mimic the large‐scale centrifuge. The material for this experiment was sourced from Culture‐C (Table I) and filtered at 200 LMH.

In a typical mAb harvest process, the unit operation subsequent to centrifugation is depth filtration. If an accurate centrate mimic was to be developed, it had to show equivalent filtration properties to that of the centrate obtained at scale. Figure 5 shows the pressure and turbidity profiles generated during the operation of the X0HC depth filter provided with two feeds: the BTPX‐305 centrate and the centrate from the preparative CSD approach calibrated against the established RSD scale‐down tool. The CSD centrate showed pressure and turbidity profiles that closely matched those of the BTPX‐305. This indicated that the CSD centrate had similar filtration properties to the BTPX‐305 centrate, confirming utility of the preparative CSD.

**Figure 6:**
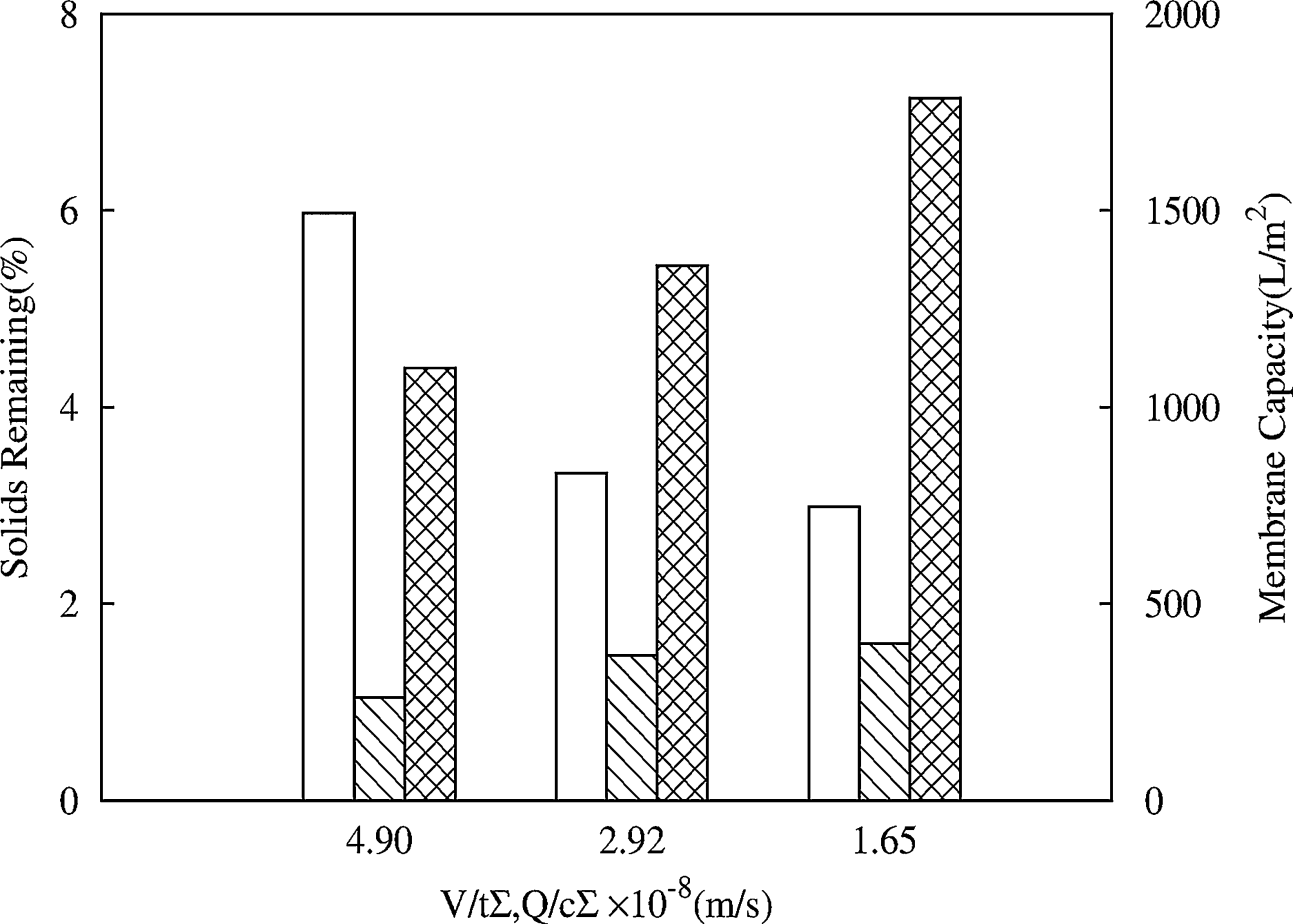
Comparison of solids remaining post centrifugation (□), and filter capacities of 0.1–2.0 μm X0HC depth filter (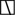) and 0.2 μm SHC sterile filter (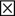). Data obtained from preparatory CSD centrate mimicking the LAPX‐404 at a range of centrifugal conditions (Table II). All centrates and filtrates were generated from processing Culture‐D (Table I).

Having established that the CSD can be used to prepare large quantities of sheared material for integrated process studies, the impact of the combination of centrifugal and depth filtration operating conditions on the performance of the primary recovery train was examined. Figure 6 shows the solids remaining and the X0HC and SHC capacities for centrates mimicking the LAPX‐404 centrifuge at a range of conditions (Table II). Unsurprisingly, the increase in capacities of both X0HC and SHC are inversely related to the levels of solids remaining.

**Figure 7:**
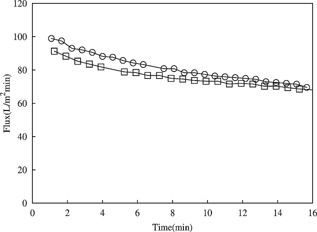
Comparison of 0.2 μm SHC sterile filter flux decline at 10 psi. Feed was from a 0.1 to 2.0 μm depth filter (X0HC) operated at 200 LMH (□) and 50 LMH (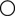). Both filtrates generated from processing Culture‐E (Table I) using the preparatory CSD mimicking LAPX‐404 at a *V*/tΣ of 2.92 × 10^−8^ (m/s) (Table II).

When the impact of flux on the X0HC depth filter and subsequent SHC sterile filtration operations was examined, it was found that with the given centrate an increase in flux from 50 to 200 LMH had no significant effect on the capacity of the depth filter (50 LMH: 407 L/m^2^, 200 LMH: 435 L/m^2^). The flux decline profiles (Fig. 7) for the SHC suggested that X0HC filtrates created at the two fluxes examined (50 and 200 LMH) had similar fouling propensities.

Subsequent studies focused on the impact of cell culture conditions on the performance of a harvest operation comprising centrifugation, depth filtration, and sterile filtration. The cell cultures examined represented differing extremes of harvest challenge; a difficult to harvest material and a relatively facile to recover broth. The former was from a bioreactor producing a high cell density, low viability cell culture whereas the latter was characterized by relatively low cell density and high cell viability.

**Figure 8:**
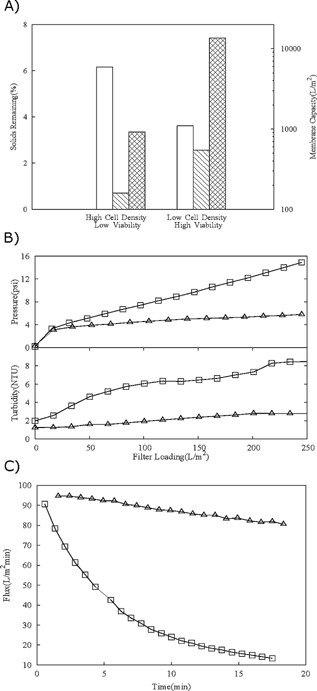
Comparison of process performance at different stages of the primary recovery sequence. Feed streams were sourced from two cell cultures representing difficult to harvest material (Culture‐F) and easy to harvest material (Culture‐G). The culture properties for this are listed in Table I. (**A**) (□) Post centrifugation mimicking LAPX‐404 at a *V*/tΣ of 2.92 × 10^−8^ (m/s) (Table II); (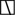) capacity of 0.1–2.0 μm depth filter (X0HC) at 200 LMH; (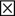) capacity of 0.2 μm sterile filter (SHC) at 10 psi. (**B**) Comparison of pressure and turbidity profiles generated operating 0.1–2.0 μm depth filter (X0HC) processing centrate obtained via the preparative methodology using Culture‐F (□) and Culture‐G (△). Centrate generated to mimic the LAPX‐404 operated at *V*/tΣ of 2.92 × 10^−8^ (m/s). (**C**) Comparison of 0.2 μm sterile filter (SHC) flux decline for Culture‐F (□) and Culture‐G (△). The filtrate was generated through conditioning both cultures using the preparative methodology to mimic the LAPX‐404 at *V*/tΣ of 2.92 × 10^−8^ (m/s) and processing the centrate through a 0.1–2.0 μm depth filter (X0HC) at 200 LMH.

It was shown that processing difficult to harvest cultures negatively impacted the ability of the centrifuge to remove solids (Fig. 8A). This form of material is challenging for harvest operations as there is a high solid content, leading to the centrifuge bowl filling up rapidly and requiring a high frequency of discharge. Another challenge was the low viability of the material. Such broths will contain large populations of cell debris which are small in size and have a lower density than viable cells. The removal of such cell debris by centrifugation is difficult and these particles often end up in the centrate resulting in a poor separation. However, the easy to harvest broth has a lower solid content and a greater population of large viable cells, which facilitates separation. Furthermore, it may be expected that high cell viability may lead to healthy cells with intact cell walls and consequently, more resistant to shear damage within the centrifuge feed zone. This would be expected to yield a centrate of high clarity and fewer cell debris particles being passed on to subsequent filtration processes hence requiring much lower capacities when processing easy to harvest cultures (Fig. 8A). Figure 8B shows the X0HC pressures and turbidity profiles for centrates derived from both culture materials. Upon filtration, the centrate material from difficult harvest conditions showed significant increases in both the pressure and turbidity profiles (Δ15.5 psi, Δ8.7 NTU) compared to that of the easy to harvest material (Δ6.0 psi, Δ2.8 NTU). This can be directly attributed to the higher levels of smaller debris in the centrate of the high cell density culture causing a more rapid rate of pore blockage.

As noted in Figure 8A, processing of the difficult to harvest culture yielded lower SHC capacities compared to the easy to harvest culture. This was attributed to the larger amount of small particles carried through from the high cell density culture which in turn lead to a more rapid rate of pore blockage and flux decline (Fig. 8C).

## Conclusion

This work examines the impact of primary recovery process parameters and cell culture conditions on the performance of subsequent centrifugal harvest and both depth and sterile filtration as a process sequence. In order to characterize the process sequence, the ability to prepare adequate volumes of sheared samples was necessary. This study commenced by establishing the conditions of operation for a preparative device based on capillary shear to create levels of shear damage similar to the industrially accepted standard RSD used routinely for scale‐down studies.

Experiments were conducted to determine the flow rates through the preparative CSD to generate equivalent levels to the shear developed in the standard RSD which has already been shown to match shear levels found in the feed zone of industrial disk‐stack centrifuges. The centrates generated were then filtered. In the process of filtration, equivalent turbidity and pressure profiles were observed to those obtained when filtering centrate generated from a large‐scale disk stack centrifuge.

The preparative methodology was then applied to explore how culture conditions impact primary recovery. One feed was from a culture producing high cell densities and low viabilities. This feed had a negative effect on the performance of a typical bioprocess sequence reducing the centrifuge capability to clear solids and also reducing the capacity of subsequent depth and sterile filtration processes. Altering the centrifugal operating conditions to produce centrates with lower solids remaining yielded higher capacities of both depth and sterile filters.

The proposed preparative methodology enables the user to calibrate and confirm the operating conditions of the CSD and bench centrifuge that would allow for predictive small‐scale centrifuge and polishing filter performance. It was demonstrated that the scale‐down methodology can successfully predict large‐scale performance. This adds to the toolbox of available small‐scale models for high throughput characterization of mammalian cell culture processes. Future work should leverage the methodology developed in this publication to incorporate a wider range of cell culture broths and other models of disk‐stack centrifuges.

## Nomenclature

*a*″, *b*″: dimensionless coefficients for the linearized intermediate pore blockage model
c: correction factor to allow for flow conditions not considered in the theoretical derivation of Σ for large‐scale centrifuges
LDH_NS_: absorbance of no shear sample at 450 nm
LDH_SS_: absorbance of sheared sample at 450 nm
OD*_f_*: optical density of feed at 600 nm
OD*_o_*: optical density of well spun sample at 600 nm
OD*_s_*: optical density of supernatant at 600 nm
*P*_max_: predicted capacity at 10 psi for constant flow operations (L/m^2^)
*Q*_0_: initial volumetric filtrate flow rate (L/h)
*Q*_ds_: flow rate of pilot scale disk‐stack centrifuge (L/h)
*r*_1_: inner radius of centrifuge discs (m)
*r*_2_: outer radius of centrifuge discs (m)
*R*_1_: radius to sample surface in an individual tube (m)
*R*_2_: radius to sample base in an individual (m)
S: solids remaining in processed samples (%)
t: filtration time (h)
*t*_lab_: laboratory centrifugation time (h)
V: total filtrate volume per filter area (L/m^2^)
*V*_lab_: volume of suspension in individual tubes (L)
*V*_max_: predicted maximum volume filtered per unit area for constant pressure operations (L/m^2^)
x: fraction of overall centrifugation time for deceleration phase
y: fraction of overall centrifugation time for acceleration phase
z: number of discs in disc‐stack centrifuge
∑_ds_: equivalent disk stack centrifuge settling area (m^2^)
∑_lab_: equivalent laboratory centrifuge settling area (m^2^)
ω: angular velocity (s^−1^)
